# Chiral covalent organic cages: Construction and chiral functions

**DOI:** 10.1002/smo.20240038

**Published:** 2025-03-25

**Authors:** Si‐Dan Guo, Tianyu Jiao, Dong‐Sheng Guo, Kang Cai

**Affiliations:** ^1^ College of Chemistry State Key Laboratory of Elemento‐Organic Chemistry Frontiers Science Center for New Organic Matter Nankai University Tianjin China; ^2^ Department of Chemistry National University of Singapore Singapore Singapore; ^3^ College of Chemistry State Key Laboratory of Elemento‐Organic Chemistry Key Laboratory of Functional Polymer Materials (Ministry of Education) Frontiers Science Center for New Organic Matter Collaborative Innovation Center of Chemical Science and Engineering Nankai University Tianjin China; ^4^ Xinjiang Key Laboratory of Novel Functional Materials Chemistry College of Chemistry and Environmental Sciences Kashi University Kashi China

**Keywords:** chirality, covalent organic cages, enantioselectivity, porous materials, synthetic strategy

## Abstract

Covalent organic cages (COCs) are three‐dimensional organic molecules with permanent cavities, known for their ordered pore structures, excellent processability, and modular design. They have shown significant potential in applications such as gas adsorption, molecular separation, and catalysis. Introducing chiral elements into COCs results in chiral COCs with confined chiral cavities, which endows them with unique chiral functions and expands their application prospects. This review summarizes the research progress on chiral covalent organic cages, focusing on strategies for incorporating chiral elements, the structures and synthesis methods of representative chiral COCs, and advancements in their chiral functions. Additionally, we provide perspectives on future research directions. We hope this review will inspire further interest and creativity among researchers in the field of chiral molecular cages, leading to the development of materials with unique structures and functions.

## Graphical abstract

Chiral covalent organic cages (COCs) with chiral confined cavities possess unique properties and have extensive application value in various fields. This review summarizes the research progress on chiral COCs, including synthetic strategies, methods to introduce chirality, and major applications. At the end of this review, future research directions are anticipated.

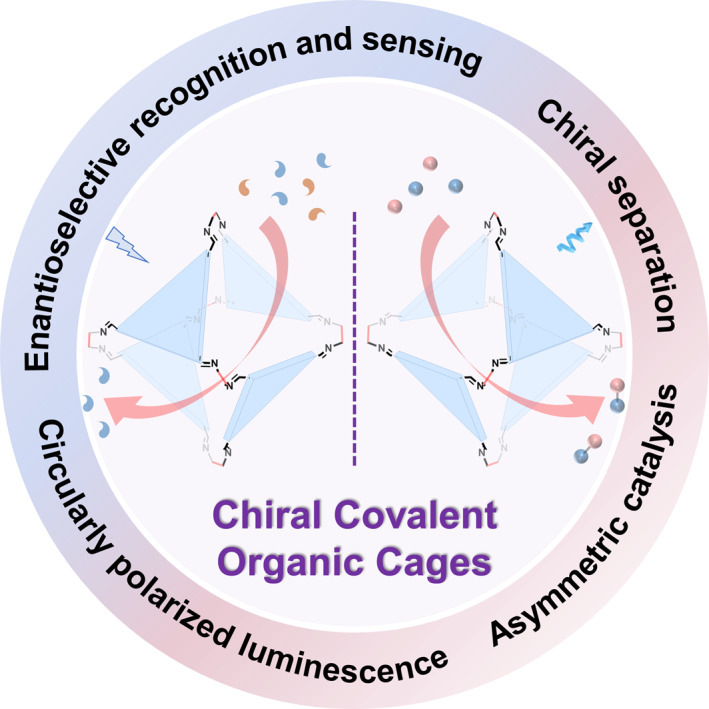

## 1 INTRODUCTION

Over the past few decades, compounds with defined pores have consistently attracted the research interest of chemists and materials scientists. From classical inorganic porous materials like zeolites to the more contemporary and continuously popular metal‐organic frameworks (MOFs),[^1^, ^2^, ^3^] covalent organic frameworks (COFs),[^4^, ^5^, ^6^, ^7^] and porous organic polymers (POPs),[^8^, ^9^, ^10^] these materials are characterized by their open, permanent, interconnected pores and large surface areas. As a result, they exhibit significant practical applications in areas such as gas adsorption and separation, energy storage and conversion, water purification, heterogeneous catalysis, molecular sensing and detection, and drug delivery.[^11^, ^12^, ^13^, ^14^, ^15^, ^16^, ^17^, ^18^]

In addition to these extended porous materials with permanent voids, recent years have seen a growing focus on discrete hollow cage molecules, such as metal‐organic cages (MOCs) and covalent organic cages (COCs). This interest stems from the fact that MOCs and COCs not only possess all the advantages of large surface areas, porosities, and open pore channels but also, as discrete molecular species, they exhibit good solubility in common solvents. This solubility enables their solution dispersibility and processability, which is a distinct advantage over insoluble extended porous frameworks. As an increasing number of molecular cages with diverse structures and properties are synthesized, they have shown immense potential in various fields, including molecular recognition, sensing, separation, catalysis, and more.[^19^, ^20^, ^21^, ^22^, ^23^, ^24^] For more detailed discussions on the research progress of MOCs and COCs, one can refer to several excellent review articles published in recent years.[^25^, ^26^, ^27^, ^28^, ^29^]

On the other hand, chirality is widespread in nature, and biological systems exhibit a strong preference for homochirality. This phenomenon makes the study of the synthesis, detection, separation, biological activity, and function of chiral compounds become a topic of common interest across various disciplines including chemistry, pharmacology, materials science, and environmental science.^[30]^ Introducing chiral elements into molecular cages yields chiral confined pore structures, which in turn bring about chiral effects and functions.[^31^, ^32^, ^33^, ^34^] Therefore, the syntheses and functions of chiral cages are important directions in the field of molecular cage research.

This review primarily focuses on the research progress in chiral COCs. We will discuss the construction strategies of chiral COCs, including (i) the direct construction of chiral COCs using building blocks with different types of chiral elements (point chirality, axial chirality, planar chirality), (ii) the construction of chiral COCs using non‐chiral building blocks, and (iii) post‐synthetic modifications of existing chiral COCs to obtain new chiral COCs. It is noteworthy that the most effective method for constructing chiral cages currently relies on imine condensation, which means that a significant portion of the chiral cages discussed in this review are imine‐based. Although chiral cages formed through non‐imine condensation methods represent a smaller subset, we have included this category to broaden the scope of the review, as it represents an important area for future development in chiral cages. Additionally, we will provide an overview of the research progress on the chiral functions of COCs, such as chiral recognition and sensing, chiral separation, asymmetric catalysis, and circularly polarized luminescence. We conclude with suggestions for future directions for chiral COCs in contemporary chemistry and materials science.

## 2 SYNTHETIC STRATEGIES OF CHIRAL COCs

For the construction of chiral COCs, chiral elements need to be incorporated into the COCs structures. The most widely exploited approach is to use chiral molecules as building blocks, where the chirality of the molecular cages is directly determined by the chiral building blocks. For instance, point chiral molecules like (*R*,*R*)‐/(*S*,*S*)‐1,2‐cyclohexanediamine, axially chiral 1,1′‐binaphthyl‐2,2′‐diol (BINOL), planar chiral triaminotribenzotriquinacene (TBTQ), and helicenes with helical chirality have all been used to construct chiral COCs. The second approach involves using non‐chiral building blocks which during the cage formation process undergo symmetry breaking or conformational restriction resulting in inherently chiral COCs. This method avoids the use of chiral precursors, but the formed COCs are usually racemic mixtures requiring further chiral resolution to obtain enantiopure COCs. The third approach involves the post‐modification of already existing chiral COCs to alter their structures and obtain new chiral COCs with different properties. In this section, we will introduce the three strategies, respectively, and showcase the representative chemical structures of different chiral COCs.

### 2.1 Construction of chiral COCs by using different types of chiral building blocks

#### 2.1.1 Chiral COCs based on building blocks with point chirality

Point chiral compounds are the most abundant source of chiral elements. Using point chiral diamines to react with non‐chiral polyaldehyde compounds in condensation reactions is the most common method for synthesizing chiral imine cages. By varying the structures of chiral diamines and non‐chiral polyaldehydes, a wide range of chiral COCs with different sizes and geometries can be conveniently constructed. For example, the reaction of chiral diamines with tri‐, tetra‐, penta‐, or hexa‐aldehyde compounds, combined with different stoichiometric ratios in the condensation reaction, can significantly alter the geometry of the resulting imine cages.

**FIGURE 1:**
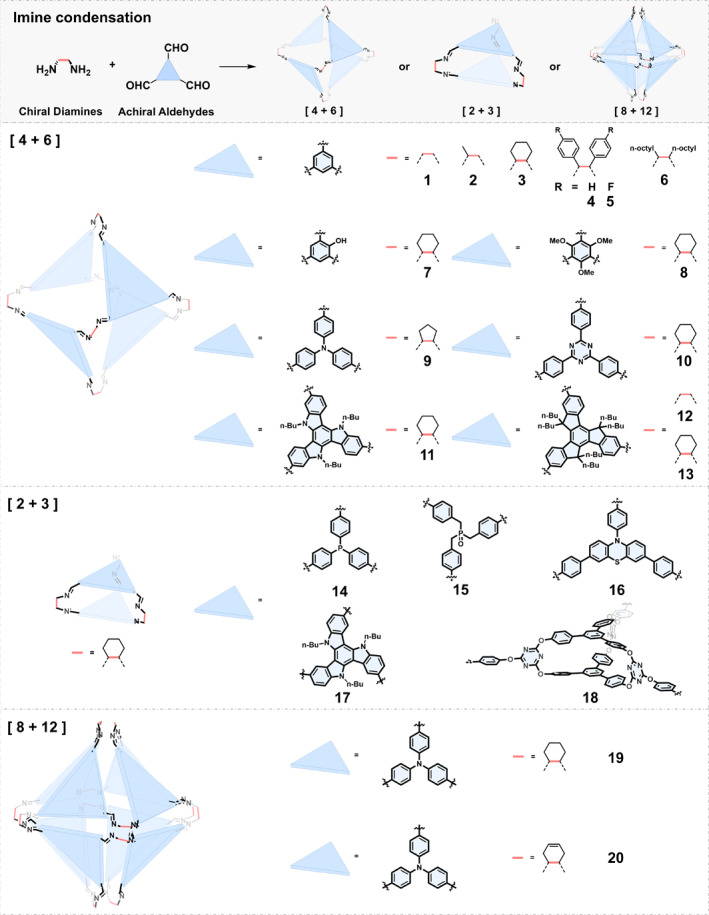
Construction of chiral organic cages by condensation reactions between chiral diamines and trialdehydes.

*Condensation between Chiral Diamines and Trialdehydes*: As shown in Figure 1, three types of cages can be formed by imine condensation of chiral diamines and trialdehydes namely [4 + 6], [2 + 3], and [8 + 12] cages.

In 2009, Cooper et al.^[35]^ reported the 2:3 condensation between 1,3,5‐triformylbenzene and 1,2‐ethylenediamine (cage 1), 1,2‐propylenediamine (cage 2), or (*R*,*R*)‐1,2‐diaminocyclohexane (cage 3), respectively, leading to the formation of three tetrahedral symmetric [4 + 6] imine cages 1–3. The products were isolated directly as crystalline solids and characterized by single‐crystal X‐ray diffraction. Notably, the different vertices had significant effects on the crystalline packing structures and the connectivity of the cage voids in cages 1‐3, resulting in distinct porosities. Among them, cage 3 exhibited unique permanent 3D pore networks, resulting in a high specific surface area (*SA*_BET_ = 624 m^2^ g^−1^). This work initiated a surge of interest in imine cages as a novel class of crystalline porous materials. Subsequently, Cooper et al. synthesized a series of [4 + 6] imine cages (4–6) by varying the molecular structures of different diamines.[^36^, ^37^] They conducted a systematic and in‐depth study on how different vertices affected the physical properties, crystalline packing structures, and porosities of these imine cages.

Inspired by Cooper's work, many researchers have selected different triformyl compounds to condense with diamines to synthesize [4 + 6] imine cages of various sizes and properties. For example, in 2020, Zhao et al.^[38]^ synthesized a series of new [4 + 6] cages with nanoscale pores and confirmed their good water permeation and complete salt rejection through experiments and simulations. Cage 3, 7, and 9 represented different water and ion transport capabilities due to their different pore window sizes, structural rigidity, hydrophilicity, and ability to form a network of interconnected channels. Thus, the effect of slight structural changes on chiral COCs is significant.

While imine cages typically exhibit insufficient stability against hydrolysis, Banerjee et al.^[39]^ found that when 2,4,6‐trimethoxy‐1,3,5‐triformyl benzene was used to assemble with (*R*,*R*)‐1,2‐diaminocyclohexane, the resulting chiral cage 8 demonstrated high chemical stability against hydrolysis under both acidic and basic conditions, probably due to the favorable hydrophobic and electronic effects of methoxy groups.

Yuan and coworkers^[40]^ exploited electron‐deficient 1,3,5‐tris‐(4‐formyl‐phenyl)triazine to react with (*R*,*R*)‐1,2‐diaminocyclohexane, resulting in a chiral imine cage 10, which has a very large cavity (∼2070 Å^3^) and a high BET surface area of 1181 m^2^ g^−1^. Cao et al. investigated the assembly of *C*_3_‐symmetric triazatruxene and truxene derivatives with (*R*,*R*)‐1,2‐diaminocyclohexane or 1,2‐ethylenediamine to synthesize [4 + 6] cage 11–13.[^41^, ^42^] Notably, symmetry breaking occurred during the assembly process of the triazatruxene/truxene, resulting in the planar chirality of these building units. Consequently, cage 12 could theoretically exist in five different stereoisomeric forms from a mathematical standpoint. However, only one pair of *T*‐symmetry enantiomers was obtained and separated by chiral HPLC.

The condensation of diamines with triformyl compounds can lead to the formation of not only [4 + 6] imine cages but also [2 + 3] type imine cages. For example, in 2021, Ding et al.^[43]^ reported a [2 + 3] cage 14 containing triphenylphosphine, which was used as a cage ligand to catalyze homogeneous hydroformylation reactions. Huang and Dai synthesized [2 + 3] chiral cage 15 using the flexible tris(4‐formylphenyl)phosphate and (*R,R*)‐1,2‐diaminocyclohexane, and the single crystal structure showed that two polar P=O units point towards the cage cavity.^[44]^ Cage 15 was able to selectively capture CO_2_ and exclude CH_4_ due to size‐matching and polarity effects. Mukherjee et al.^[45]^ synthesized a [2 + 3] chiral imine cage 16 by the self‐assembly of (*S*,*S*)‐1,2‐diaminocyclohexane with an triformyl substituted phenothiazine. Moreover, when Cao et al.^[42]^ changed the substitution position of the formyl group on the triazatruxene, they also obtained a [2 + 3] imine cage 17. Recently, Chen et al.^[46]^ used a triformyl substituted molecular cage as 3D tri‐bladed propeller‐shaped building blocks to synthesize a pair of enantiopure [2 + 3] molecular cages 18 by imine condensation.

In rare instances, condensation between diamines and trialdehydes may also lead to the formation of [8 + 12] imine cages. For example, Cooper et al.^[47]^ found that when tris(4‐formylphenyl)amine was assembled with (*R*,*R*)‐1,2‐diaminocyclohexane or (*R*,*R*)‐1,2‐cyclohex‐4‐enediamine, large [8 + 12] octahedral cage 19 and cage 20 were obtained, respectively, instead of forming conventional [4 + 6] cages. It is evident that the formation of the [8 + 12] cage requires the participation of 20 components in the reaction, which is generally thermodynamically unfavorable. Consequently, this remains the only reported instance to date.

**FIGURE 2:**
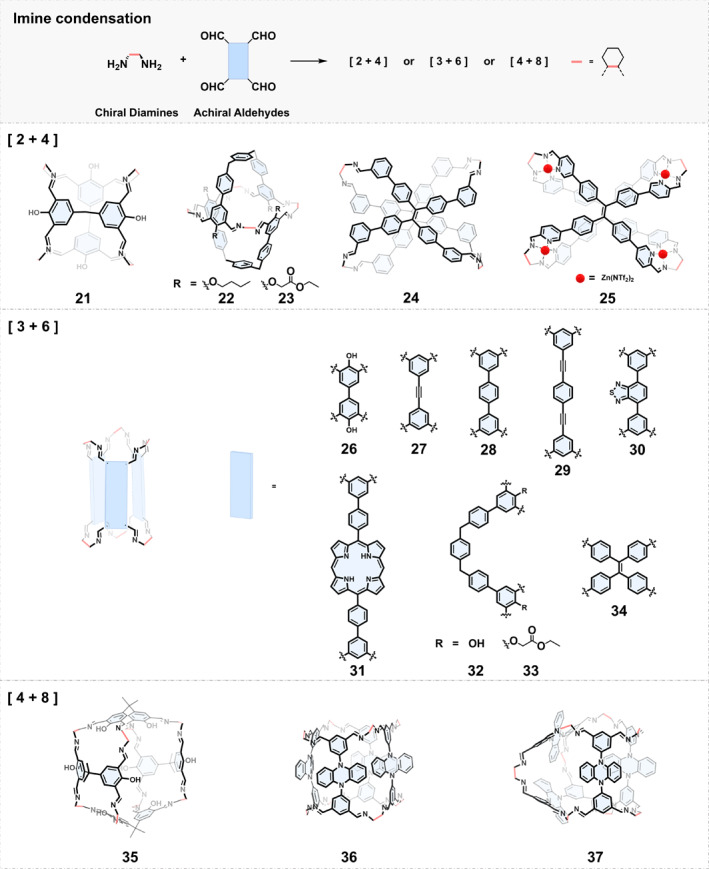
Construction of chiral organic cages by condensation reactions between chiral diamines and tetraaldehydes.

*Condensation between Chiral Diamines and Tetraaldehydes*: When using tetraaldehydes and diamines for condensation to construct imine cages, the three common possible outcomes are [2 + 4], [3 + 6], and [4 + 8] cages (Figure 2). One typical example came from Huang et al.^[48]^ In 2020, they investigated the assembly between three structurally different tetraaldehydes and (*S,S*)‐ or (*R*,*R*)‐1,2‐diaminocyclohexane in the presence of EtOH/H_2_O/MOH (M = Na, K, and Cs). Interestingly, [2 + 4] cage 21, [3 + 6] cage 26, and [4 + 8] cage 35 were obtained, respectively. They found that the formation of different types of cages was mainly determined by the angles of the two benzene ring planes adjacent to the building blocks.

Another important finding was reported by Cooper and coworkers in 2016.^[49]^ They utilized three tetraaldehyde precursors with different linker lengths to condense with (*R*,*R*)‐ or (*S*,*S*)‐1,2‐diaminocyclohexane affording three chiral tubular covalent cages 27–29. The three tubular cages have triangular windows at each end. The single crystal structures indicated that the cages with different chirality can be self‐assembled to form one‐dimensional supramolecular nanotubes by stacking alternately from neighboring window to window, further creating 3D diamondoid pillared porous networks. Therefore, this work demonstrates a general strategy for synthesizing [3 + 6] cages with tubular cavities.

Jiang and coworkers^[50]^ employed a tetraaldehyde derivative of benzo[c][1,2,5]thiadiazole to assemble with (*R*,*R*)‐1,2‐diaminocyclohexane which led to the formation of a chiral [3 + 6] tubular cage 30. The thiadiazole units in the cavity can coordinate with Pd^2+^, which can further support ultrafine Pd nanoparticles to form a composite catalyst to catalyze organic reactions. Lately, they introduced porphyrin rings into the [3 + 6] tubular molecular cage and obtained cage 31.^[51]^ The single crystal structure revealed that the adjacent molecular organic cages accumulated into a 1D supramolecular nanotube, and further accumulated into a 3D porous supramolecular framework. Cage 31 was found to exhibit excellent heterogeneous photocatalytic performance and can effectively promote the photo‐oxidation coupling reaction of various primary amines. Additionally, tetraphenylethylene‐based tetraaldehyde has also been utilized to construct [2 + 4] cages (24, 25) and [3 + 6] cages 34, showing good fluorescence and circularly polarized luminescence.[^52^, ^53^, ^54^, ^55^]

Relatively flexible tetraaldehydes have also been used to construct imine cages, and their assembly process is more easily influenced by substituents adjacent to the aldehyde groups. Li et al.^[56]^ designed a series of U‐shaped tetraaldehydes with different substitutes between two aldehydes to assemble with (*R*,*R*)‐ or (*S*,*S*)‐1,2‐diaminocyclohexane leading to the formation of either achiral [2 + 4] (22, 23) or chiral [3 + 6] (32, 33) cages. They suggested that different substituents provide different hydrogen bonding patterns and thus mediate the conformational orientation of aldehyde groups, resulting in the formation of cages with different geometry and chirality.

The condensation between tetraaldehydes and diamines can also lead to the formation of [4 + 8] type cages.^[57]^ Recently, Cui and coworkers^[58]^ reported the assembly between a tetraaldehyde derivative of 5,10‐di(3,5‐diformylphenyl)‐5,10‐dihydrophenazine and (*R,R*)‐ or (*S,S*)‐1,2‐diaminocyclohexane. Notably, two homochiral porous [4 + 8] imine cages, 36 and 37, were generated simultaneously, and the ratio of the two was affected by temperature. The single crystals of cages were obtained by recrystallization of crude products using different binary solvents. It was confirmed that cage 37 had a Johnson‐type *J*_*26*_ structure, and cage 36 had a tetragonal prismatic structure.

**FIGURE 3:**
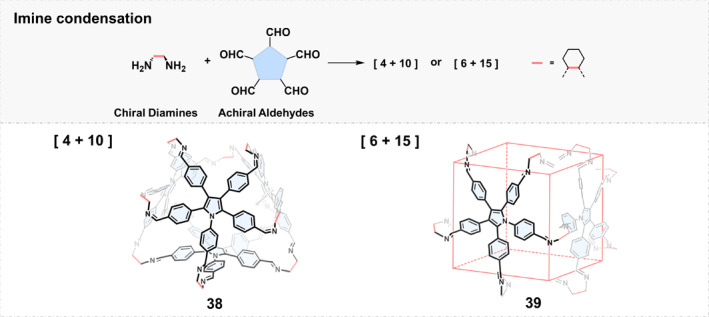
Construction of chiral organic cages by condensation reactions between chiral diamines and pentaaldehydes.

*Condensation between Chiral Diamines and Pentaaldehydes*: Pentaaldehyde precursors for imine condensation are relatively difficult to design and synthesize, and the resulting imine cage structures are also not obvious to predict. Cao and coworkers^[59]^ suggested using the graph theory to help predict the symmetry and topology of the imine polyhedra. Accordingly, they designed and synthesized a new type of facial construction unit based on pentaphenylpyrrole.^[60]^ 1,2,3,4,5‐penta (4‐phenylaldehyde)pyrrole (PPP) that has five rotatable phenyl groups and exhibits either clockwise or anticlockwise 2D rotational patterns due to the steric hindrance between the benzene rings. The assembly between PPP and enantiopure 1,2‐diaminocyclohexane was highly dependent on the choice of solvent, and the template effect of solvent seriously affected the cage structure during the assembly process. When trimethylbenzene was used as the solvent, cage 38 was assembled showing a truncated tetrahedron structure. When the solvent was replaced by 1,2‐dichloroethane, it was assembled into a truncated rhombohedron cage 39 (Figure 3). Both cages have large cavity volumes of up to 864 and 1926 Å^3^, respectively.

**FIGURE 4:**
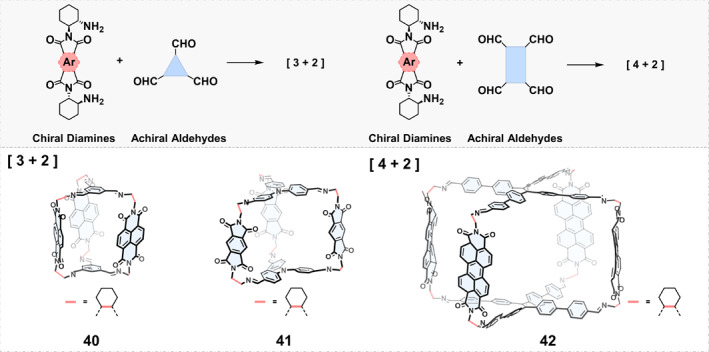
Construction of chiral organic cages by condensation reactions between other chiral diamines and multialdehydes.

*Assembly of Other Chiral Diamines and Multialdehydes*: Condensing (*R*,*R*)‐ or (*S*,*S*)‐1,2‐diaminocyclohexane with planar arylene dianhydrides in a 1:1 ratio can produce chiral arylene diimides with two amino groups, which can serve as precursors for imine condensation with various polyaldehydes to synthesize chiral imine cages. In 2017, Wasielewski et al.^[61]^ reported a [3 + 2] cage 40 by employing naphthalene diimide (NDI) diamine to react with 1,3,5‐triformylbenzene (Figure 4). Electron hopping and charge separation between three NDI units were found in cage 40. In 2019, Zhang and coworkers^[62]^ obtained a pair of enantiopure [4 + 2] chiral imine cages 42 via condensation between a perylene diimide (PDI)‐derived chiral diamine and a tetraaldehyde precursor. Cage 42 with large hydrophobic cavities (ca. 1.5 × 0.9 × 0.8 nm^3^) can encapsulate planar polycyclic aromatic hydrocarbons and demonstrated the photocatalytic activity of the cage in visible‐light‐driven Smiles rearrangement reactions. Recently, Liu and coworkers^[63]^ designed and synthesized a pair of [3 + 2] chiral cage 41, which displayed enhanced fluorescence and CPL in solid states when crystalized with tris(pentafluorophenyl)borane, since tris(pentafluorophenyl)borane can serve as “brakes” to inhibit the rotation of phenyl groups of the cage in the crystalline state.

**FIGURE 5:**
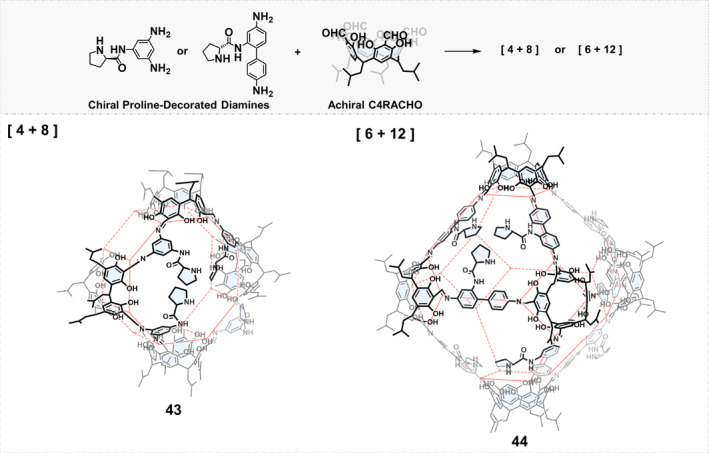
Construction of chiral organic cages by condensation reactions between chiral proline‐decorated diamines and tetraformyl‐functionalized calix[4]‐resorcinarene.

Macrocyclic multialdehydes and aromatic diamines were also exploited to synthesize chiral imine cages. As shown in Figure 5, Yuan et al.^[64]^ synthesized two chiral proline‐decorated diamine building blocks with different shapes and reacted them with tetraformyl‐functionalized calix[4]‐resorcinarene to obtain [4 + 8] cage 43 and [6 + 12] cage 44. The precise introduction of chiral proline endowed the cages with excellent enantioselective catalytic properties.

#### 2.1.2 Chiral COCs based on axially chiral building blocks

**FIGURE 6:**
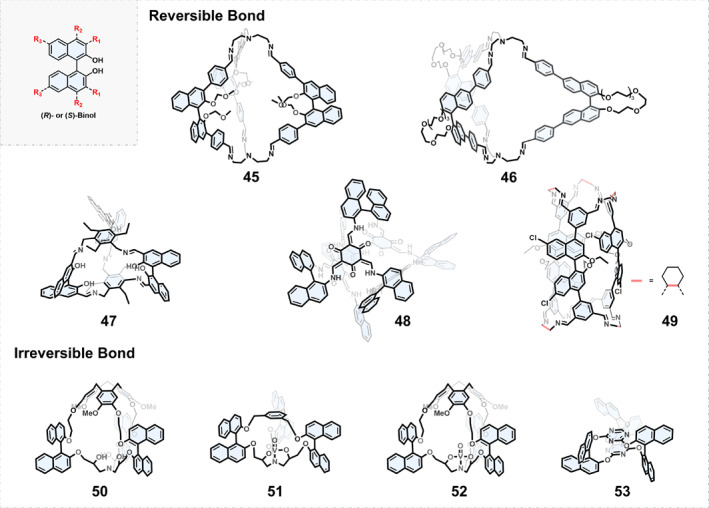
Construction of chiral organic cages using axially chiral BINOL as building blocks.

Axial chiral molecules acquire chirality from their spatial arrangement around the axis of rotation. The simplest axial chiral molecule is allene. Chemists used appropriately substituted allene as a rigid chiral axis to construct helical chiral molecular cages with different topologies.[^65^, ^66^, ^67^, ^68^] However, in most cases, the best option for researchers when choosing axially chiral building blocks is 1,1′‐binaphthyl‐2,2′‐diol (BINOL). The backbone of BINOL can be functionalized at different positions to produce a variety of axially chiral building blocks for the construction of chiral COCs either by dynamic covalent chemistry or irreversible bond formation (Figure 6).

*BINOL‐derivatized Chiral COCs Based on Dynamic Covalent Chemistry*: In 2021, Huang and Li^[69]^ reported a pair of enantiopure [2 + 3] (*S*)‐ and (*R*)‐BINOL‐based imine cages 45 by the condensation between an (*S*)‐ or (*R*)‐BINOL‐derived dialdehyde and tris(2‐aminoethyl)amine. Cage 45 showed a *C*_3_ symmetric topology in which three BINOL units form a narrow chiral cavity. The authors further reduced it to a more stable amine cage and explored its potential application in enantioselective recognition. Similarly, Song et al.^[70]^ introduced benzaldehyde units to the *R*_3_ position of BINOL units to prepare a pair of enantiopure [2 + 3] cages 46. Mobian^[71]^ synthesized a smaller [2 + 3] cage 47 which has six OH groups oriented towards the interior of the cavity, providing more opportunities for chiral recognition, post‐modification, and coordination chemistry. It is worthy to note that Sun and Wang^[72]^ utilized binaphthyl‐2,2′‐diamine to react with 2,4,6‐trihydroxy‐1,3,5‐triformyl benzene and obtained an enantiopure cage 48. The keto‐enol tautomerism‐induced intramolecular hydrogen bonds endowed cage 48 with excellent chemical stability.

Synthesis of heterochiral molecular cages from two or more chiral building blocks is rarely reported. By pairwise assembly of the enantiomers of a binaphthol‐based tetraaldehyde and enantiopure diaminocyclohexane, Wang and Jiang^[73]^ obtained a pair of enantiopure heterochiral organic molecular cages. The corresponding self‐assembly kinetics were studied by luminescence and nuclear magnetic resonance (NMR) spectroscopy, and the enantioselective recognition mechanism in the assembly process was found. The enantiomers of BINOL‐based tetraaldehyde precursors were assembled with diaminocyclohexane in the opposite chiral configuration to form heterochiral cage 49.

*BINOL‐derivatized Chiral COCs Based on the Formation of Irreversible Bonds*: In addition to using dynamic covalent chemistry, BINOL derivatives can also be employed in irreversible nucleophilic substitution reactions to construct chiral COCs. Although the yields may be relatively lower, these chiral COCs exhibit significantly better chemical stability. Typically, the two hydroxy groups of BINOL compounds can undergo nucleophilic substitution reactions with precursors that have three reactive halogen atoms, resulting in [2 + 3] type chiral COCs. For example, a cyclotriveratrylene (CTV) derivative reacted with BINOL to afford a chiral COC 50 in 25% yield.^[74]^ The chiral cavity of cage 50 can perform stereoselective recognition of glucose and mannose derivatives. Similarly, by modifying the precursor structure, researchers have further synthesized chiral COCs 51–53.[^75^, ^76^]

#### 2.1.3 Chiral COCs based on building blocks with helical chirality

**FIGURE 7:**
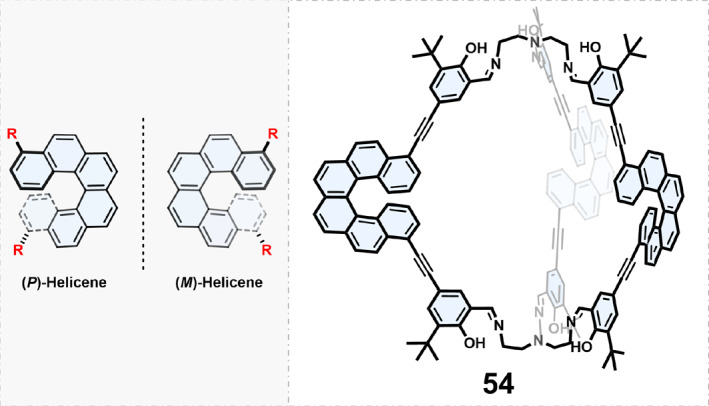
Construction of chiral organic cages using building blocks with helical chirality.

Helicenes are non‐planar helical compounds formed from ortho‐fused benzene rings with a unique structure and inherent chirality. Helicene derivatives can be synthesized in various ways and are expected to be used as chiral building blocks to construct chiral COCs.^[77]^ However, only a few reports have been published to date.[^78^, ^79^] Qiu and coworkers^[78]^ used enantiopure helicene‐based dialdehydes as the building blocks to obtain chiral covalent organic cage 54 through the condensation reaction with tris(2‐aminoethyl)amine (Figure 7). The organic cages exhibited a twisted structure, with three helicene units arranged in a propeller‐like fashion, resulting in a triple‐stranded helical structure. Further studies confirmed that cages had the ability of enantioselective recognition of a series of aromatic racemates.

#### 2.1.4 Chiral COCs based on building blocks with planar chirality

**FIGURE 8:**
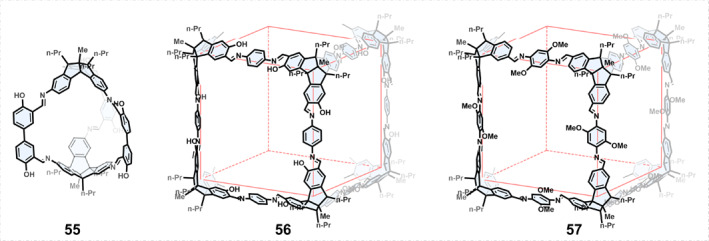
Construction of chiral organic cages using building blocks with planar chirality.

Compounds with stable planar chirality are relatively uncommon. Mastalerz^[80]^ developed a methodology to synthesize *C*_3_‐symmetric, planar chiral triaminotribenzotriquinacene (TBTQ) derivatives, and employed them to construct chiral COCs. By using racemic TBTQ trialdehydes as the precursors, racemic mixtures of homochiral [3 + 2] cage 55 were obtained because of narcissistic chiral self‐sorting in solution (Figure 8). However, a small portion of heterochiral cages also coexisted in the solution because of social self‐sorting. Exclusive social self‐sorting can be achieved by exploiting the solubility differences between homochiral and heterochiral cages.

In 2021, Mastalerz^[81]^ further discovered a class of large cubic [8 + 12] salicylimine cage compounds 56 during the assembly of racemic *C*_3_‐symmetric TBTQ‐tris(salicylaldehydes) and 1,4‐phenylenediamine. Theoretically, there should be 23 possible [8 + 12] cage stereoisomers, but only three isomers have been generated because of chiral self‐sorting (racemic cages: heterochiral cages = 45:55 in CD_2_Cl_2_). The enthalpy and entropy of the system are conducive to the high selectivity of chiral self‐classification and the formation of highly symmetric cage products. The authors exploited the differences in solubility to isolate the heterochiral cage with a yield of 83%, although the energy differences between the homochiral and heterochiral cages were small.

Mastalerz explored interesting superstructures of interlocked cages using non‐covalent interactions. By changing the reaction conditions, cage 57 or dimeric and trimeric catenanes could be formed.^[82]^ The authors used various dialdehydes with TBTQ triamine reactions under different conditions, and found that when the substituents were methoxy or thiomethyl it was conducive to the formation of chain alkanes. This may be due to the weak interactions between the substituents and the solvophobic effects.

### 2.2 Construction of inherent chiral COCs using achiral building blocks

**FIGURE 9:**
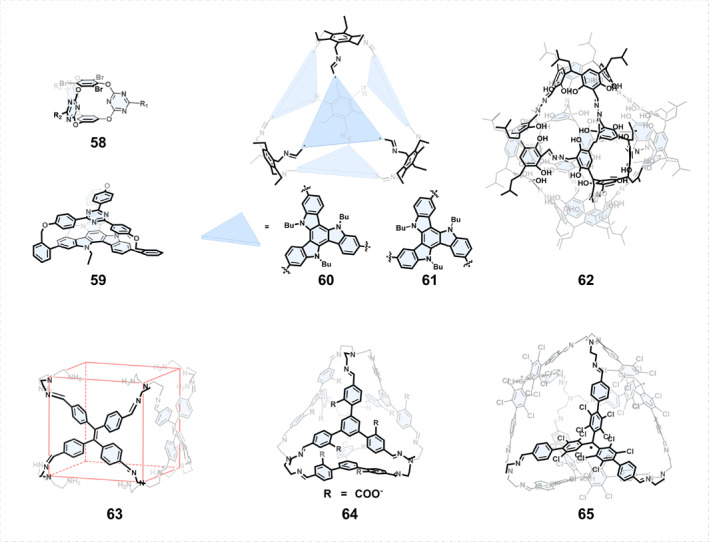
Construction of chiral organic cages by achiral building blocks.

In addition to transferring the chirality of chiral building blocks to covalent organic cages, achiral building blocks can also be used to construct inherently chiral COCs (Figure 9). In 2022, Wang^[83]^ achieved this by substituting different groups to gradually reduce the symmetry of triazine cages, ultimately obtaining a series of inherently chiral COC derivatives as racemic mixtures, such as cage 58.

Building blocks with *C*_3h_ symmetry are achiral due to the presence of a symmetry plane. However, once these achiral planar building blocks are used to construct COCs, the symmetry plane will be disrupted, resulting in stable planar chirality and imparting chirality to the COCs. For example, triazatruxene is a typical *C*_3h_ symmetric molecule.^[59]^ In 2024, Zhang et al.^[84]^ reported the synthesis of racemic [2 + 3] cage 59, which showed excellent CPL (|*g*_lum_| = 2.1 × 10^−3^) and TADF properties. Cao et al.^[85]^ utilized triazatruxene trialdehydes to synthesize cages 60 and 61. Interestingly, all five possible diastereoisomers of cage 60 existed when assembled using *meta*‐formyl‐substituted triazatruxene and 1,3,5‐triaminomethyl‐2,4,6‐trithylbenzene (Tri‐NH_2_). However, only a pair of *homo*‐directional enantiomeric cage 61 was generated when *para*‐substituted constitutional isomer was used.

Another molecular cage 62 with inherent chirality was quantitatively produced by the reaction of tetraformylresorcin[4]arene and tetrahydrazone.^[86]^ The reason for the inherent chirality of the cubic nanocage 62 (*O* symmetry) was the directional arrangement of the hydrazone groups and the rigid structure stabilized by hydrogen bonds, giving the cage a 2283 Å^3^ cavity.

Another type of achiral building block is the propeller‐like molecule such as tetraphenylethylene (TPE), which usually has multiple freely rotating phenyl groups, and thus exhibits nonplanar plus (*P*) and minus (*M*) rotation configurations due to intramolecular steric hindrance. Because of fast racemization under room temperature, the propeller‐like molecules commonly cannot show stable chirality. However, when being incorporated into COCs, the rotation of phenyl groups will be restricted, leading to stable chirality. For example, Wang and Cao^[87]^ prepared four [6 + 8] face‐rotating chiral imine cages using a TPE derivative. Notably, each cage had different orientational and rotational configurations, and the (6*M*)‐cage 63 was shown in Figure 9.

In 2021, Li et al.^[88]^ reported the synthesis of [4 + 4] cages by the aqueous‐phase imine condensation of a trisbenzaldehyde precursor bearing substituent units and tris(2‐aminoethyl)amine. In the case of cage 64, the presence of noncovalent interactions such as CH⋅⋅⋅π interactions or hydrogen bonds limited the rotation of phenyl units, and the four propeller‐shaped trisformyl residues in the tetrahedral cage had the same helical configuration (*P* or *M*), endowing the molecular cage with homochirality. Multiple imine covalent bonds enhanced the stability of the cage, so cage 64 was fairly stable in water. The stereoselectivity of the assembly was also successfully controlled by chiral templates. In 2021, the same group^[89]^ reported a [4 + 4] homochiral radical cage 65 and confirmed the existence of a pair of homochiral enantiomeric cages by single crystal structure. The intramolecular interactions such as CH⋅⋅⋅π interactions and hydrogen bonds resulted in chiral self‐sorting during the cage formation. It was found that the enantiomers still showed very stable homochirality after chiral HPLC separation, probably because the steric hindrance generated by chlorine atoms prevented the flipping of phenyl units within the cage.

### 2.3 Construction of chiral COCs by post‐synthetic modification of chiral COCs

Post‐synthetic modification of chiral COCs provides an alternative important route to obtain new chiral COCs. Post‐synthetic modification can not only significantly alter the structural rigidity, chemical stability, and cavity properties of COCs, but also give rise to COCs with different physicochemical properties, such as solubility, crystallinity, porosity, and guest binding capabilities.^[90]^

**FIGURE 10:**
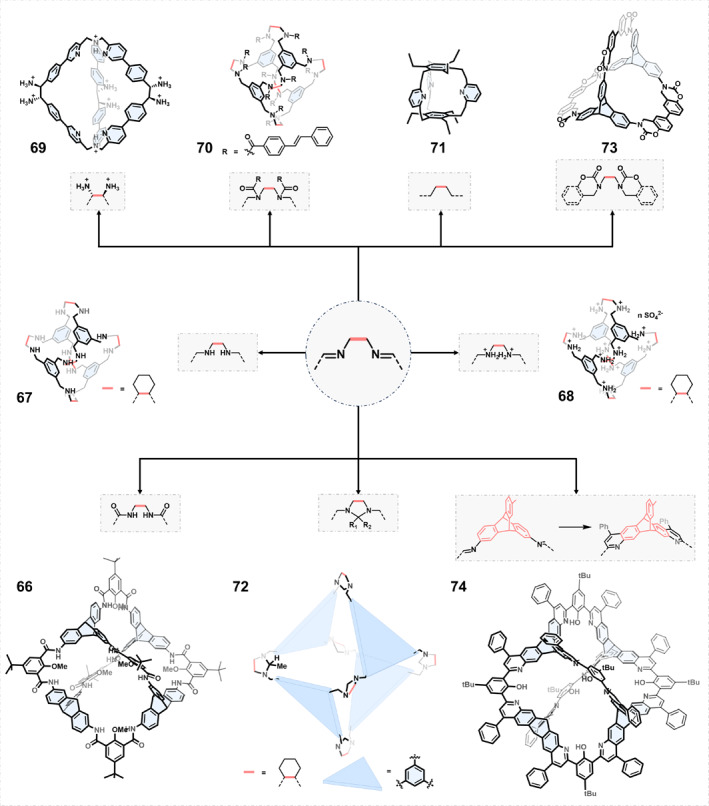
Construction of chiral organic cages by post‐synthesis modification.

Given that imine cages are the most widely synthesized (chiral) COCs, research on the post‐synthetic modification of COCs has primarily focused on imine cages. The stability of the imine cage is influenced by the reversibility of the imine bond. As shown in Figure 10, a variety of post‐synthetic modification strategies can be used to convert imine bonds into other functional groups in situ, thereby improving the chemical stability. The REDOX reaction, which oxidizes imines to amides or reduces imines to amines to improve the stability of COCs, is the most straightforward strategy. Amide cages have the same shape‐persistence as imine cages but also have the chemical stability that imine cages lack. In 2019, Mastalerz^[91]^ obtained a shape‐persistent [4 + 6] amide cage 66 via Pinnick oxidation of an imine cage. The formation of 12 amide bonds greatly increased the chemical stability of the cage, allowing it to maintain chemical robustness in the pH range from −1 to 14.5. The high stability of cage 66 allowed further functional modification under harsh conditions such as nitration and bromination.

The stability and solubility of the cage can both be improved by reducing the imine cage to an amine cage. However, amine cages no longer have shape persistence and often appear to have a nonporous or microporous structure due to the flexible C‐N bond. The confinement effect of cage 67 with a flexible cavity could stabilize Pd nanoclusters and prevent nanoclusters from aggregating, resulting in excellent solubility of Pd@cage 67 hybrids.^[92]^ The cage salt 68 was obtained after further protonation.^[93]^ The cavity of the cage salt was filled with water molecules, and the proton transfer could be promoted by confining the water molecules. The proton conductivity can reach up to 10^−3^ S cm^−1^.

Using flexible amine cages as intermediates, a series of dodecaamide cages can be obtained from amidation. The synthesis of cage 70 required only a two‐step reaction to introduce 12 functional arms.^[94]^ By changing the different R groups, a series of 12‐arm organic building blocks could be produced, which could be used to prepare porous amorphous materials such as microporous dendrimers.

Besides, the rigidity of the amine cages can be restored by various methods. Cooper^[95]^ reported a strategy called post‐synthetic “tying” of a flexible amine cage. The shape persistence of the amine cage with both chemical stability was achieved by the formation of aminal via the reaction of diamine and aldehyde or ketone. Subsequently, a strategy of protect‐functionalize‐deprotect in confined space was proposed, which could realize a series of cage compounds with different cavity sizes.^[96]^ Due to the presence of steric hindrance, the reaction of amine cage and acetone only reacted at one vertex. In the solid state, the remaining five unfunctionalized diamine groups reacted with gaseous formaldehyde to form five five‐membered rings. After selective hydrolysis of isopropyl, the exposed diamines could be further functionalized by carbonyl groups such as formaldehyde (cage 72), acetaldehyde, and propionaldehyde.

Another method, similar to the above, was to react the reduced amine cage with N,N′‐carbonylbisimidazole at room temperature to obtain a carbamate cage.^[97]^ Cage 73 had high acid‐base stability and could be maintained at 100°C. Cage 73 was a permanently porous structure that enabled large amounts of CO_2_ adsorption at 273 K and 1 bar (58 cm^3^ g^−1^; 11.5 wt%).

Zonta et al.^[98]^ transformed an unstable imine cage into a hydrolytically stable chiral cage 69 through the [3,3]‐sigmatropic Diaza‐Cope rearrangement. The single crystal structure showed that cage 69 had intrinsic porosity and the formation of the C‐C bonds enhanced the stability of cage 69, which showed a high stability at low pH. Another strategy for the construction of hydrocarbon cages was based on reduced amine cages. The imine cage was converted to the hydrocarbon cage 71 by three steps, namely reduction, nitration, and Overberger reaction.^[99]^ The synthesis of the hydrocarbon cage based on dynamic imine bonds not only shortened the traditional synthesis route but also increased the overall yield.

Mastalerz^[100]^ converted the imine cage into a quinoline cage 74 through the Povarov reaction of a [4 + 6] salicylimine cage with phenylacetylene. The shape‐persistent cage 74 had excellent chemical stability in the pH range from −1.9 to 15.2 and did not break down when heated to 350°C. Due to the presence of numerous nitrogen atoms, cage 74 exhibited unique acidochromic properties and was used as a film for acid vapor detection.

## 3 APPLICATIONS OF CHIRAL COCs

Based on the formation of reversible and irreversible bonds, a large number of chiral COCs with varied structures and properties have been reported. Owing to their porosity, inherent chiral confined cavity, good solubility, and crystalline properties, chiral COCs have been widely explored as functional materials across many research fields. In this section, we summarize the research progress on the applications of chiral COCs in different areas including chiral recognition^[101]^ and sensing,^[102]^ chiral separation, asymmetric catalysis, and CPL.

### 3.1 Enantioselective recognition and sensing

The enantioselective recognition ability of chiral COCs depends mainly on the shape and size of the chiral cavity and the multi‐point non‐covalent interactions between chiral COCs and chiral substrates.

**FIGURE 11:**
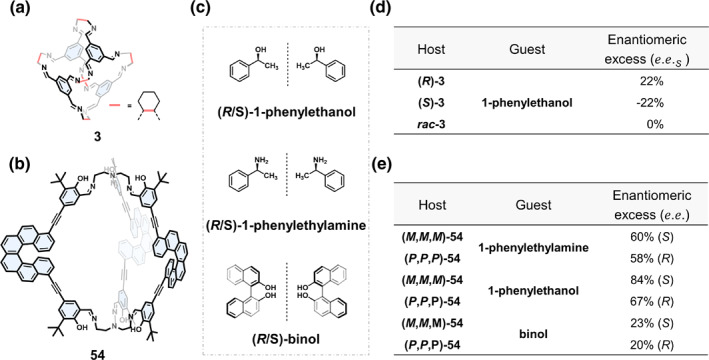
(a) Structural formula of chiral COC 3.^[103]^ (b) Structural formula of chiral COC 54.^[78]^ (c) Structural formulas of chiral enantiomers of the guests. (d) Measured enantiomeric excess of the *S* enantiomer (*e.e.*_*S*_) of 1‐phenylethanol adsorbed in cage 3 at a guest:host ratio of 2.0. (e) Measured enantiomeric excess of the enantioselective adsorption experiments of cage 54. Adapted with permission.^[78]^ Copyright 2018, American Chemical Society.

In 2014, Cooper^[103]^ demonstrated that chiral imine cage 3 was able to selectively recognize chiral enantiomers of 1‐phenylethanol. As shown in Figure 11, homochiral cage (*R*)‐3 or (*S*)‐3 could adsorb racemic 1‐phenylethanol enantiomers with an enantiomeric excess (*e.e.*) value of 22%, whereas *rac*‐3 had no enantioselective adsorption ability. Although the *e.e*. value for enantioselective adsorption is not high, this work demonstrated the potential of using chiral COCs for enantioselective discrimination of chiral compounds.

Qiu's group^[78]^ verified the enantioselective adsorption capacity of cage 54 in isopropanol solutions of several racemic compounds. As shown in Figure 11b,e, (*M*,*M*,*M*)‐54 showed excellent recognition ability for the *S*‐configuration enantiomers of 1‐phenethylamine and 1‐phenylethanol with *e.e*. values of 60% and 84%, respectively. (*P*,*P*,*P*)‐54 showed selectivity for the *R*‐configuration enantiomers and the *e.e*. values were 58% and 67%, respectively. The enantioselective adsorption of the larger molecules, such as (*R*/*S*)‐binol, was poor because of the limited cavity size.

Later on, more studies have demonstrated the chiral recognition ability of chiral COCs. For example, Li^[104]^ reported a special pair of enantiopure [1 + 3] imine cages via the assembly of a hexaformyl compound with (*S*,*S*)‐ and (*R*,*R*)‐diaminocyclohexane in water. The ^1^H NMR spectra indicated that 1,2‐epoxybutane could be enantioselectivly separated by cages. Wang and Jiang^[73]^ investigated the ability of heterochiral cage 49 to recognize a series of chiral small molecules and found that it was capable of enantioselective recognition of carvone. (*S*,*R*)‐49 showed specific chiral recognition of *D*‐carvone.

Numerous studies have demonstrated that covalent organic cages possess significant recognition and sensing capabilities for various analytes, particularly with imine cages being utilized for the fluorescent sensing of nitroaromatic compounds and metal ions.[^105^, ^106^, ^107^, ^108^] In this review, we concentrate on the unique recognition and sensing functions of chiral molecules afforded by chiral confined cavities. When the chiral recognition process can give rise to an optical signal change, chiral sensing and discrimination of enantiomeric compounds could be possible. The output of the signal can be achieved in many ways, such as electrochemical or optical signals. The optical method is easy to operate and has the characteristics of high sensitivity and rapid analysis.

Novel enantioselective potentiometric sensors were designed using 3% (*R*)‐3 and 4% cage 4 to prepare membrane electrodes, respectively.[^109^, ^110^] The Nernst response for *S*‐2‐amino‐1‐butanol was preferential with enantioselectivity coefficients of −0.98 and −1.333.

**FIGURE 12:**
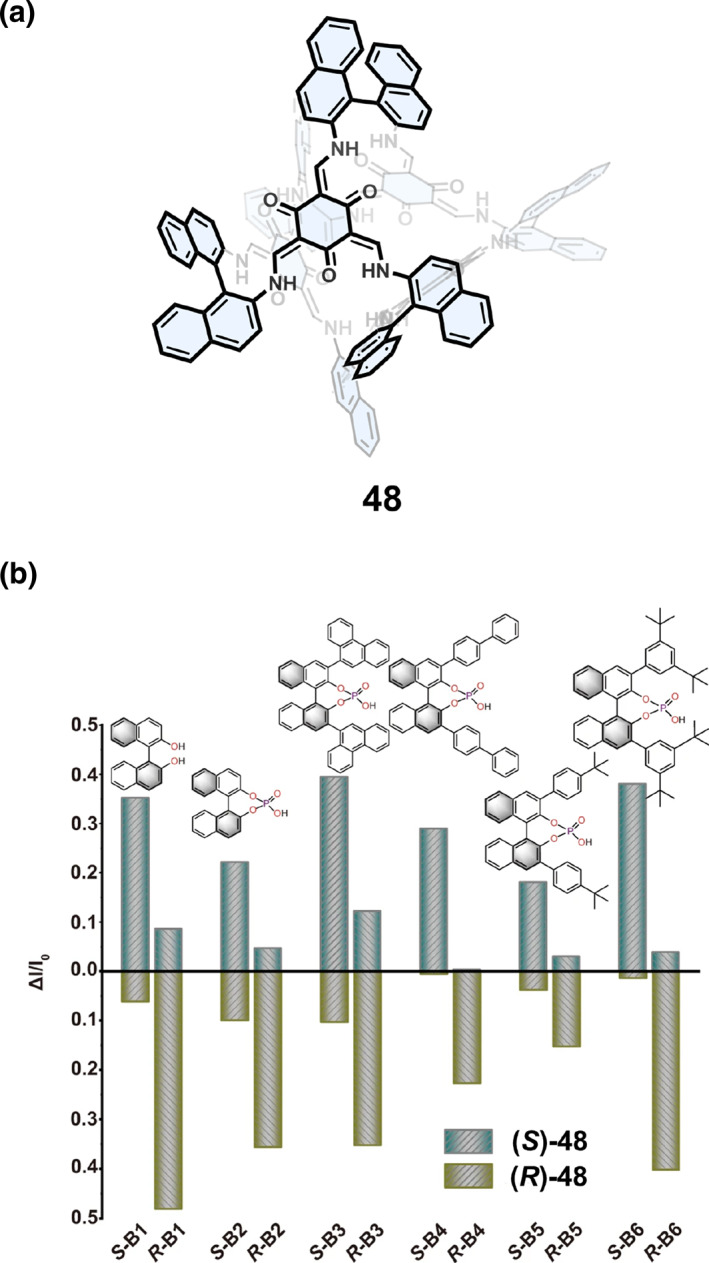
(a) Structural formula of the chiral COC 48. (b) Normalized fluorescence intensity change after adding (*R*/*S*)‐49 into the solution of axial chiral enantiomers (B1‐B6). Reproduced under terms of the CC‐BY license.^[72]^ Copyright 2022, Cui et al. published by Springer Nature.

Sun and Wang^[72]^ found that enantiopure cage (*R*/*S*)‐48 had good enantioselective recognition of a range of axial chiral aromatic racemes. As shown in Figure 12b, cage (*S*)‐48 selectively recognized *S*‐chiral substrates through the detection of fluorescence changes, especially *S*‐B4 with a selectivity of up to 98%. The experimental results showed that after five rounds of recycling, the sensing capability was still maintained at ∼85% and the detection limit was 0.597 μM. Molecular dynamics (MD) simulations and DFT calculations indicated that both the host and guest complexes showed close packing. Cage (*S*)‐48/*S*‐B4 showed back‐to‐back stacking, whereas cage (*S*)‐48/*R*‐B4 showed cross stacking. The energy transfer between cage (*S*)‐48 and *S*‐B4 was more efficient because the excited energy transfer of cage (*S*)‐48/*S*‐B4 had a greater electron coupling than that of cage (*S*)‐48/*R*‐B4.

### 3.2 Chiral separation

Separation of enantiomers is of great significance in both pharmaceutical and biological fields. Due to their chiral recognition ability, chiral COCs have often been used as chromatographic stationary phases in capillary columns for gas chromatography separation. In 2015, Yuan et al.^[111]^ prepared a silica capillary column by static coating using chiral COC (*R*)‐3 diluted with polysiloxane OV‐1701 as the stationary phase. The results confirmed that compared with the commercial Chirasil‐L‐Val and *β*‐DEX 120 columns, this stationary phase had superior chiral separation ability in gas chromatography. It effectively separated a wide range of racemic compounds including chiral alcohols, diols, amines, alcohol amines, esters, ketones, ethers, halohydrocarbons, organic acids, amino acid methyl esters, and sulfoxides. Cooper et al.^[112]^ also demonstrated that the chiral COC (*R*)‐3 stationary phase had the ability to separate a range of racemes such as linear alkanes, chiral purities, and amines.

**FIGURE 13:**
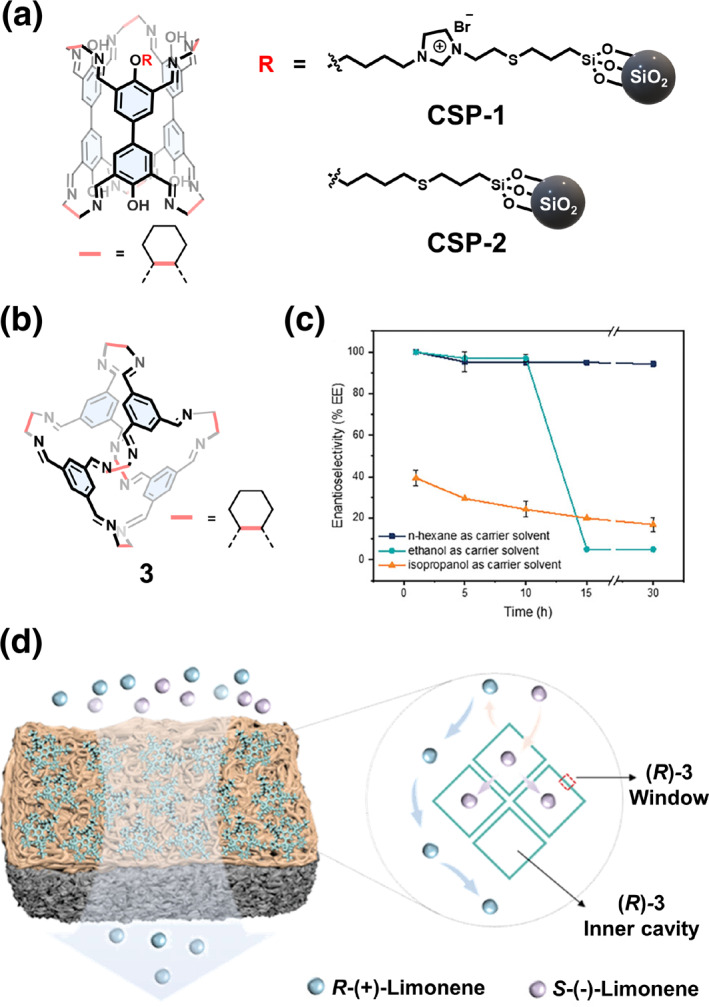
(a) Structural formulas of the CSP‐1 and CSP‐2.^[113]^ (b) Structural formulas of the chiral COC 3. (c) The change in enantioselectivity with time for the separation of limonene racemates prepared in different organic solvents. (d) Schematic of the cage (*R*)‐3/PA composite membrane and the chiral recognition of the limonene enantiomers. Reproduced under terms of the CC‐BY license.^[114]^ Copyright 2024, Wang et al. published by John Wiley and Sons.

Zhang and Yuan^[113]^ used thiol‐ene click chemistry to attach chiral COC (*R*)‐26 to the surface of thiol‐functionalized silica and prepared two chiral stationary phases (CSPs) (Figure 13a). Using a typical slurry packing method, the CSPs were loaded into HPLC columns to evaluate their chiral separation performance. The results showed that the resolution effect of CSP‐1, with the cationic imidazolium spacer, was significantly greater than that of CSP‐2. This could be due to the electrostatic interaction between CSP‐1 and the analytes which favors chiral recognition.

Another approach for chiral separation involves the preparation of enantioselective membranes using chiral COCs. Wang et al.^[114]^ prepared enantioselective thin‐film‐composite membranes based on chiral COC (*R*)‐3 using polyamide (PA) as the matrix through an interfacial polymerization method (Figure 13d). Cage (*R*)‐3 selectively recognized *S*‐(−)‐Limonene molecules, while *R*‐enantiomers diffused into the matrix. The *e*.*e*. value of chiral separation was 95.2% with a high flux of 99.9 mg h^−1^ m^−2^.

### 3.3 Asymmetric catalysis

Research on chiral COCs in the field of catalysis mainly focuses on heterogeneous catalysis. Chiral cages can be used alone as catalysts[^43^, ^51^, ^62^, ^75^, ^115^, ^116^] or the composite catalysts can be prepared by encapsulating metal nanoparticles.[^45^, ^50^, ^92^, ^117^, ^118^, ^119^, ^120^, ^121^, ^122^]

**FIGURE 14:**
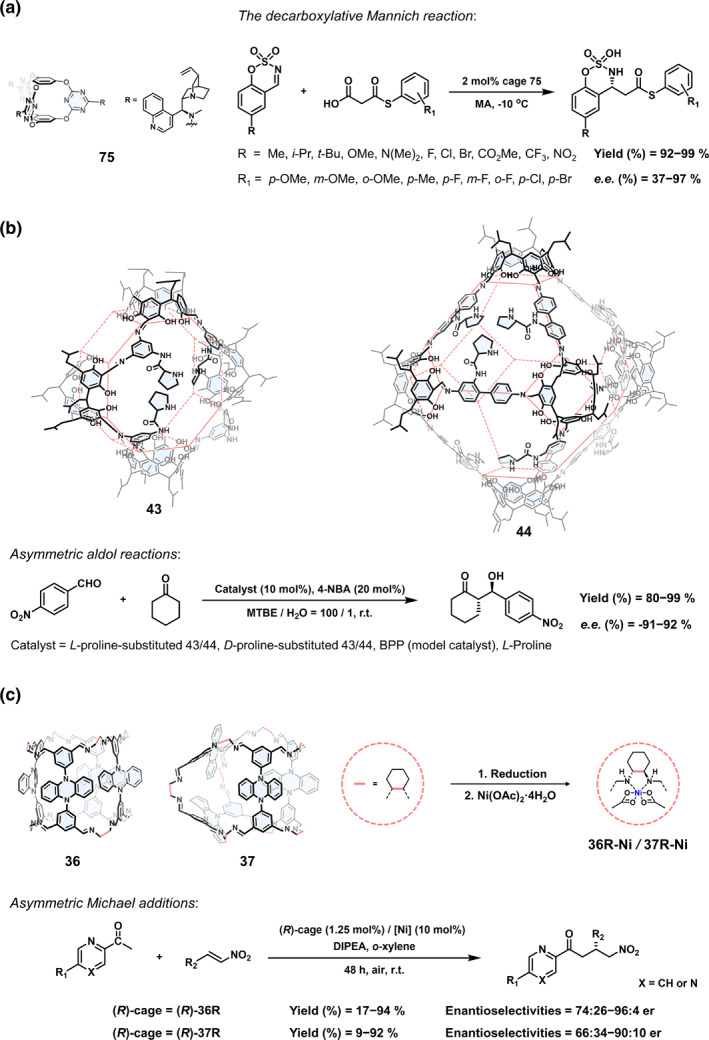
(a) Structural formula of cage 75 and the decarboxylative Mannich reaction catalyzed by cage 75.^[123]^ (b) Structural formulas of cages 43 and 44, and asymmetric aldol reactions catalyzed by chiral cages and related catalysts.^[64]^ (c) Schematic illustration of postmodifications of chiral cages 36 and 37, and asymmetric Michael additions catalyzed by (*R*)‐36R‐Ni and (*R*)‐37R‐Ni.^[58]^

Using chiral COCs for asymmetric catalysis is a highly promising yet challenging research direction. In 2021, Wang et al.^[123]^ constructed a series of chiral cage catalysts with electron‐deficient cavities. The decarboxylative Mannich reaction could be catalyzed in the π‐acidic cavity driven by anion‐π and lp‐π interactions. As shown in Figure 14a, notably, only 2 mol% of the best cage 75 catalyst could achieve near quantitative yields and up to 97% *e*.*e*. values.

In 2022, Yuan et al.^[64]^ introduced chiral proline into the building blocks and obtained chiral COCs 43 and 44 by imine condensation. The multiple proline units in the cage can mimic the chiral cavity microenvironment of enzymes. When the asymmetric aldol condensation reaction was catalyzed, it was found that the difference in the spatial distribution of chiral organic catalytic sites would affect the catalytic activity. As shown in Figure 14b, when cage 43 was used as the catalyst, the reaction rate was faster than cage 44. However, cage 44 showed higher yields and *e.e*. values. The better asymmetric catalytic performance of cage 44 may be due to the accumulation of reactants and the more effective contact between the active sites and the substrates in the chiral confined cavity.

In 2024, Cui et al.^[58]^ employed metalate chiral organic cages as a catalyst for an asymmetric Michael addition reaction. As shown in Figure 14c, the exposed diamine groups could act as bidentate ligands to coordinate with Ni(II) ions after reducing cage 36 and cage 37 to amine cages. The asymmetric Michael addition reaction of 2‐acetylazaarenes to chiral nitroalkenes catalyzed by the metalate amine cages was investigated. Cage (*R*)‐36R showed better enantioselectivity and reactivity, possibly due to its larger cavity size and open windows, which made it easier to adsorb and concentrate reactants for reaction.

### 3.4 Circularly polarized luminescence (CPL)

**FIGURE 15:**
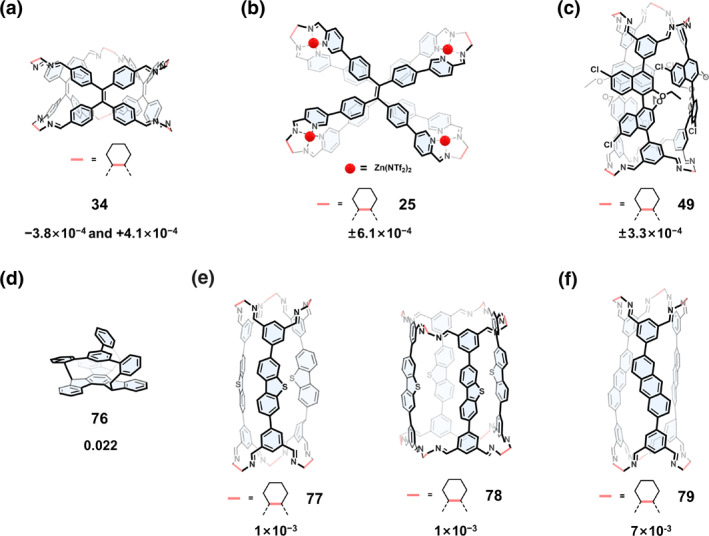
(a) Structural formula of cage 34 and the dissymmetry factor (*g*_lum_) values.^[54]^ (b) Structural formula of cage 25 and *g*_lum_.^[53]^ (c) Structural formula of cage 49 and *g*_lum_.^[73]^ (d) Structural formula of cage 76.^[124]^ (e) Structural formulas of [3 + 6] cage 77 and [4 + 8] cage 78, and the dissymmetry factor values.^[57]^ (f) Structural formula of cage 79.^[125]^

When fluorescent units are incorporated into chiral COCs, CPL materials can be obtained. As shown in Figure 15a, a pair of enantiopure chiral molecular cages 34 containing TPE units are fluorescent and CPL active.^[54]^ The dissymmetry factor (*g*_lum_) values of 3*P*‐34 and 3*M*‐34 are −3.8 × 10^−4^ and +4.1 × 10^−4^, respectively. The wavelength of metalated chiral cage 25 was obviously redshifted, and the chiral behavior of cage 25 showed different thermodynamic and kinetic aggregation (Figure 15b).^[53]^ The *g*_lum_ was calculated to be ±6.1 × 10^−4^. Heterochiral cage 49 also exhibited CPL behavior due to the presence of binaphthol fragments.^[73]^

In general, organic cages have dissymmetry factors in the range of 10^−4^‐10^−3^. Recently, Omine et al.^[124]^ synthesized racemic cage 76 by a metal‐free coupling reaction, and obtained enantiopure cage (*R*,*R*,*R*/*S*,*S*,*S*)‐76 by chiral HPLC resolution. The rigid conformation limited the molecular vibration, and the symmetry‐forbidden nature of the lowest‐energy transition caused by the *C*_3_ symmetrical structure occurs, thus exhibiting a large dissymmetry factor of the order of 10^−2^.

Recently, Li's group^[57]^ constructed [3 + 6] cage 77 by imide condensation at low concentrations using the dibenzothiophene with slightly bent geometry. In triangular tubular structures, dibenzothiophene units could bend inward or outward. The formation of intramolecular C‐H···π interactions caused [3 + 6] cage 77 to produce [4 + 6] cage 78 at high concentrations of trans‐1,2‐cyclohexanediamine. The maximum |*g*_lum_| values in both configurations were 1 × 10^−3^. The same group^[125]^ synthesized a series of tubular molecular cages with planar chirality. Due to the presence of aromatic luminescent units, all of them exhibited CPL behavior, in which cage 79 could reach 7 × 10^−3^ in methanol solution.

## 4 CONCLUSION AND OUTLOOK

With the development of synthesis methods and characterization techniques, many chiral COCs with different topologies and properties have been developed. Chiral COCs exhibit unique properties including discrete chiral confined cavities, modular synthesis and structural tunability, processability, and good solubility. Therefore, chiral COCs have wide application prospects in chiral recognition and sensing, chiral separation, and asymmetric catalysis. Despite the considerable progress made in the study of chiral COCs, several challenges and opportunities remain. Here are some perspectives on the construction and application of chiral COCs:

(1) Development of New Dynamic Covalent Bonds: The error‐correction mechanism inherent in dynamic covalent chemistry simplifies the synthesis and purification processes. Currently, most chiral cages are constructed using imine condensation. There is an urgent need to develop chiral COCs based on other types of dynamic covalent chemistry such as boronic ester condensation,^[126]^ alkene/alkyne metathesis,[^127^, ^128^] and so on.
(2) Exploration of New Chiral Building Blocks: The variety of chiral building blocks available for COC synthesis is currently limited. Commonly used building blocks include diaminocyclohexane for center chirality and BINOL for axial chirality. Expanding the family of chiral COCs through the development of additional chiral building blocks would significantly enhance their diversity and application potential.
(3) Creation of Water‐Soluble Chiral COCs: While chiral COCs have demonstrated potential in chiral recognition, there is a lack of research on enantioselective recognition in aqueous environments. Water‐soluble molecular cages could better utilize hydrophobic cavities and offer great potential for biomedical applications.^[129]^
(4) Chiral COCs in Chiral Separation: Currently, commercial chiral separation columns primarily utilize various types of chiral molecules—such as amino acids, cellulose, cyclodextrins, and chiral crown ethers—as chiral selectors immobilized on the stationary phase.[^130^, ^131^, ^132^, ^133^, ^134^, ^135^, ^136^, ^137^, ^138^] However, the inherent enantiomer discrimination capabilities of these chiral selectors often are quite limited.[^139^, ^140^, ^141^] By enhancing the chiral discrimination ability of single‐site chiral selectors, we can significantly improve the efficiency of chiral separation per unit length of the chiral column. Chiral COCs, being soluble, crystalline, and processable porous materials, have been extensively studied and applied in the adsorption and separation of gases and organic molecules. With their multiple chiral sites, well‐organized chiral confined cavities, and diverse structural possibilities, chiral COCs exhibit superior enantiomer discrimination capabilities compared to traditional chiral selectors like cyclodextrins or chiral crown ethers. Additionally, the cost of producing chiral COCs derived from imine condensation is relatively low. Consequently, chiral COCs hold great potential as a new generation of chiral selectors for chiral stationary phases and chiral separation membranes with promising commercial applications. While there has been some relevant research in this area, further exploration and optimization are still needed.
(5) Chiral COCs for Asymmetric Catalysis: The rich chiral sites and tunable chiral cavity environments of chiral COCs are somewhat analogous to enzymes. Therefore, using the chiral cavities of COCs directly for supramolecular asymmetric catalysis, or coordinating catalytic active metals with chiral COCs for asymmetric catalysis, are promising pathways for achieving efficient enzyme‐like asymmetric catalysis. There remains significant exploration potential in this area.
(6) Unlike polymeric and macromolecular carriers or inorganic nanomaterials, COCs exhibit uniform compositions, precise molecular structures, well‐defined confined cavities, and established host‐guest binding stoichiometries. These characteristics confer distinct advantages for drug delivery including improved batch reproducibility and delivery accuracy. Their enclosed cavities not only enhance the effective encapsulation of drug molecules but also thermodynamically and kinetically reduce the likelihood of drug leakage. When designed with stimuli‐responsive release mechanisms, COCs can function as highly effective drug delivery carriers. Moreover, the absence of heavy metals in COCs enhances their biocompatibility and reduces toxicity compared to metal‐organic cages and inorganic nanocarriers, making them particularly advantageous for biological applications.[^142^, ^143^] Given that living organisms are complex, multi‐level homochiral systems, increasing attention is being paid to the influence of chiral factors in materials on biological functions.[^144^, ^145^, ^146^, ^147^, ^148^] The precise chemical composition and structure of chiral COCs render them ideal platforms for studying the effects of chiral carriers on various biological barriers and their subsequent biological impacts. Currently, research in this area is limited, suggesting significant potential for future investigations.

In summary, chiral COCs offer a versatile and attractive topic for further exploration in various scientific and industrial applications. Addressing the outlined challenges and opportunities will pave the way for further advancements and broader utilization of chiral COCs in multiple fields.

## CONFLICT OF INTEREST STATEMENT

The authors declare no conflicts of interest.

## Data Availability

Data sharing is not applicable to this article as no new data were created or analyzed in this study.
